# Expectancy or Salience?—Replicating Senders’ Dial-Monitoring Experiments With a Gaze-Contingent Window

**DOI:** 10.1177/00187208231176148

**Published:** 2023-05-21

**Authors:** Yke Bauke Eisma, Ahmed Bakay, Joost de Winter

**Affiliations:** 2860 Delft University of Technology, the Netherlands

**Keywords:** distributed attention, supervisory control, attentional processes, eye movements, replication study, peripheral vision

## Abstract

**Introduction:**

In the 1950s and 1960s, John Senders carried out a number of influential experiments on the monitoring of multidegree-of-freedom systems. In these experiments, participants were tasked with detecting events (threshold crossings) for multiple dials, each presenting a signal with different bandwidth. Senders’ analyses showed a nearly linear relationship between signal bandwidth and the amount of attention paid to the dial, and he argued that humans sample according to bandwidth, in line with the Nyquist–Shannon sampling theorem.

**Objective:**

The current study tested whether humans indeed sample the dials based on bandwidth alone or whether they also use salient peripheral cues.

**Methods:**

A dial-monitoring task was performed by 33 participants. In half of the trials, a gaze-contingent window was used that blocked peripheral vision.

**Results:**

The results showed that, without peripheral vision, humans do not effectively distribute their attention across the dials. The findings also suggest that, when given full view, humans can detect the speed of the dial using their peripheral vision.

**Conclusion:**

It is concluded that salience and bandwidth are both drivers of distributed visual attention in a dial-monitoring task.

**Application:**

The present findings indicate that salience plays a major role in guiding human attention. A subsequent recommendation for future human–machine interface design is that task-critical elements should be made salient.

## INTRODUCTION

In the 1950s and 1960s, John Senders conducted a number of experiments on visual attention distribution (Senders, 1964; Senders et al., 1966; for a summary, see Senders, 1983). Participants were presented with a panel displaying four or six dials. Each dial featured a pointer that moved in an unpredictable manner, and participants were instructed to press a hand switch whenever any of the pointers exceeded a fixed angular threshold value on either side.

In Senders’ experiments, the presented pointer signal consisted of multiple frequencies, each with random phase shifts, making the pointer movement appear random to the human observer. Furthermore, each pointer was driven by a signal with a different bandwidth. Bandwidth, expressed in Hertz (Hz) or radians per second (1 Hz corresponds to 2π radians per second), describes the highest frequency contained in the signal. Informally, a pointer with low bandwidth can be described as, on average, slow-moving, and it will cross the threshold angle relatively infrequently. In contrast, a high-bandwidth pointer will cross the threshold value more often.

Senders proposed various mathematical models for predicting attention distribution in dial-monitoring tasks (Senders, 1983; for a review, see Eisma et al., 2020). One of the simpler models, called the Periodic Sampling Model, suggests that the sampling rate of a dial is proportional to its bandwidth (Senders, 1964). This means that dials with higher bandwidths receive more attention than those with lower bandwidths. This model is based on the Nyquist–Shannon sampling theorem, which states that a signal must be sampled at a minimum of twice its bandwidth to be accurately reconstructed. A more complex model developed by Senders is the Conditional Sampling Model, which posits that human observers distribute their attention based on bandwidth and the observed pointer angle at the moment the dial was last sampled (Senders et al., 1966, 1983; for a similar model, see Sheridan, 1970).

Senders’ research had a significant impact on later studies of human attention distribution, particularly in human–machine interaction tasks such as driving (Du et al., 2022; Horrey et al., 2006; Lemonnier et al., 2020; Yamani et al., 2018), flying (Steelman et al., 2011; Wickens et al., 2003), health care tasks (Grundgeiger, Hohm et al., 2022; Grundgeiger, Michalek et al., 2022), and interacting with robotic automation (Wickens et al., 2015). According to Wickens’ SEEV model of human attention (Wickens & McCarley, 2008), bandwidth, as conceptualized and operationalized by Senders, is one of the key factors that influence where observers are likely to direct their attention (the other factors are salience, effort, and value). Specifically, the bandwidth of a task variable enables human operators to form expectations about how often a particular task region should be sampled. If prior experiences showed that a certain task area needs to be addressed frequently (due to its higher bandwidth), then the operator is likely to frequently attend to that task in the future as well.

A limitation of Senders’ and Wickens’ models is that they only take into account foveal vision. This means that human operators are assumed to sample one task area (e.g., dial) at a time and determine where to place the next fixation based on signal properties that are learned over time. Recently, Eisma et al. (2018) replicated Senders’ work using modern eye-tracking equipment and found a close similarity with Senders’ results. Like Senders, Eisma et al. found a nearly linear relationship between dial bandwidth and the amount of attention allocated to that dial. However, Eisma et al. also found that participants were inclined to look at a dial at moments the pointer of that dial was moving quickly. In other words, the observers’ attention distribution appeared to be governed not only by expectations, but also by salience, a component that is also part of the aforementioned SEEV model (Wickens & McCarley, 2008). This finding, in turn, led to the question of how a human can recognize a faster-moving pointer based on foveal vision alone. Eisma et al. (2018) suggested that human operators must have been able to detect the pointer-speed cues using peripheral vision in order to determine which dial to look at next. This hypothesis aligns with the common notion that peripheral vision, while not providing high visual acuity, is relatively well able to detect movement (Lappin et al., 2009; McKee & Nakayama, 1984).

In one of Senders’ lesser-known works (Senders et al., 1955), it was examined whether human observers are able to read the state of a dial using peripheral vision. In this study, participants fixated on a point in front of them and had to estimate the angle (e.g., north, northwest, and west) of dials that were placed at different horizontal eccentricities (10°, 20°, up to 80°). Based on the analysis of the percentage of reading errors as a function of dial eccentricity, Senders et al. concluded that “*an observer can discriminate among settings which differ by 45° almost perfectly even when the instrument is played as much as 40° from the line of sight*” (p. 436). In designing his dial-monitoring experiments, however, Senders rejected the possibility of participants relying on peripheral vision in his experiments: “*The instruments were mounted on panels at a separation sufficient to prevent peripheral reading of the dials* …. *The pointer was finer than would ordinarily be recommended for ease of reading but since one object was to minimize peripheral uptake of information, this was not considered to be a drawback*” (Senders, 1983, p. 41).

The question remains as to how peripheral vision affects sampling behavior. Senders manually annotated video images of the human eye to determine which dial the human operator was fixating on. Advances in eye-tracking technology in the 1970s have made it possible for researchers to use gaze-contingent windows, in which the information displayed on the screen depends on the current position of the gaze (for reviews, see Rayner, 2014; Schotter et al., 2012). In this study, we replicated Senders’ (1964, 1983) dial-monitoring research as well as a recent replication study using modern eye-tracking equipment by Eisma et al. (2018), but introduced an extra condition that uses a circular gaze-contingent window to block out the participant’s peripheral vision. Specifically, the participant’s foveal vision was restricted to a circular window that was slightly larger than an individual dial. This means that when focusing on a dial, participants could only see that specific dial, while all other dials disappeared, leaving only the gray background visible outside the circle. This research aimed to examine the effect of the gaze-contingent viewing window on participants’ ability to detect threshold crossings and distribute attention across the dials.

## METHODS

This research complied with the American Psychological Association Code of Ethics and was approved by the Human Research Ethics Committee of the TU Delft. Informed consent was obtained from each participant. A total of 33 students at the TU Delft (29 men and 4 women) participated in the experiment. Their mean age was 23.9 (*SD* = 2.4). Three participants wore glasses during the experiment.

### Apparatus

**Figure 1.**
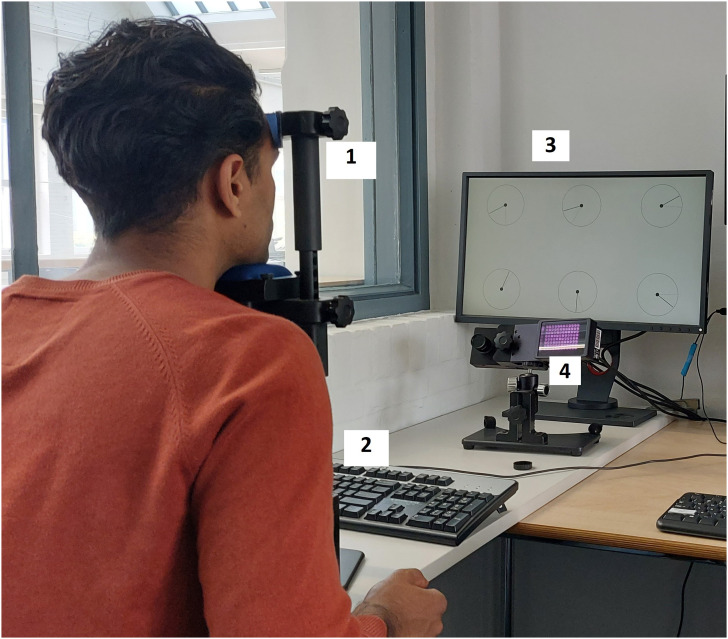
The experimental setup, including the head support (1), keyboard (2), monitor (3), and eye-tracking camera with infrared illuminator (4). A participant is performing the experiment. His head rests on the head support while his left hand (not shown) is hovering over the spacebar. The monitor shows the bank of six dials that needed to be supervised.

The eye movements of the participants were recorded binocularly with an EyeLink 1000 Plus eye tracker (SR Research) at a frequency of 2000 Hz. The stimuli were presented on a 24 inch monitor (BenQ XL2420T-B, resolution 1920 × 1080px, display area 531 × 299 mm, and refresh rate 60 Hz). Participants were asked to position their head on the head support. The distance from the eyes to the monitor was approximately 95 cm. The experimental setup is shown in Figure 1.

### Stimuli

Seven videos from Eisma et al. (2018) were used. Eisma et al. (2018) presented videos with a duration of 90 seconds, recorded at a rate of 50 frames per second, which resulted in a total of 4500 frames. For the current experiment, the videos were shortened to 60 s each by removing the last 1500 frames.

Each video featured six dials, each with a solid moving pointer and a dashed threshold line. The threshold line was fixed at a random angle that differed for each of the 42 dials (7 videos × 6 dials per video). Each dial had a diameter of 316 pixels (visual span = 5.3°). The centers of adjacent dials were 634 pixels (10.5°) apart horizontally and 658 pixels (10.9°) apart vertically, similar to Senders (1983), who reported that the dials in his six-dial experiments were separated by 12°. The six dials had the following bandwidths as per Senders (1983): 0.03, 0.05, 0.12, 0.20, 0.32, and 0.48 Hz. The mean position of the pointer signals in each of the seven videos was 0°, which corresponded to the angle of the threshold. The standard deviation of the pointer signals was 50.1°. The pointer signal was unique for each of the 42 dials.

The seven videos each had a different configuration of the dials, resulting in different levels of effort. The effort levels were previously determined based on a computer simulation that calculated the total distance the eyes needed to move in order to detect all the threshold crossings (see Eisma et al., 2018). In practice, this meant that in the lowest effort configuration, the high-bandwidth dials (0.48 and 0.32 Hz) were placed in the middle (top middle and bottom middle, respectively), while the lowest-bandwidth dials (0.03 and 0.05 Hz) were placed at the edges (bottom right and top right, respectively). For the highest effort configuration, the high-bandwidth dials were positioned at the edges (bottom right, top left), while the low-bandwidth dials were in the middle (top middle, bottom middle) (see Eisma et al., 2018 for an overview of the configuration of all dials for the seven videos).

### Gaze-Contingent Window

**Figure 2.**
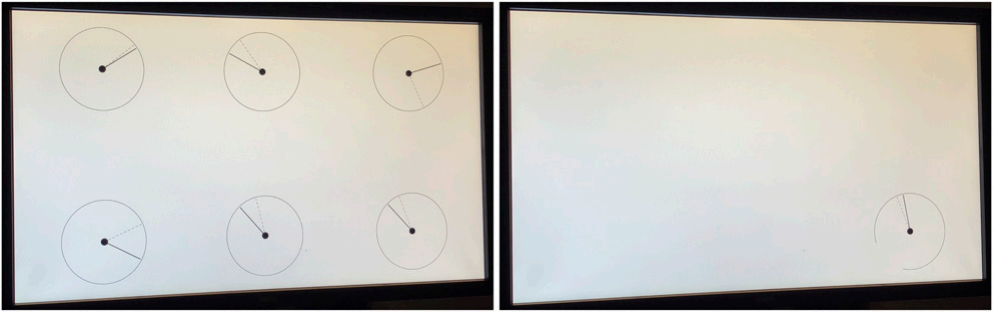
Photos of the monitor for the full-view condition (left) and the gaze-contingent condition (right). Note that the dials had a diameter of 316 pixels, while the gaze-contingent window had a diameter of 500 pixels. Hence, if looking at the very top of the dial, the bottom part of the dial was not shown (as illustrated in the right figure).

The stimuli were presented under two conditions. In one condition, the seven videos were presented in full view (as was done in Eisma et al., 2018), and in the other condition, the same videos were shown but with a gaze-contingent window that followed the participant’s gaze point. The window was circular and had a diameter of 500 pixels, corresponding to a span of 8.3°. Photos of the full-view condition and the gaze-contingent condition are shown in Figure 2. Note that the parafoveal region of the eye can be described as the area within 4.2° from the point of fixation, thus having a span of 8.4° (Sakurai, 2015). In other words, the size of the gaze-contingent window is approximately the size of the parafoveal region of the human eye. The parafoveal region has a lower density of cone photoreceptors compared to the fovea, but still has a relatively high visual acuity and plays a crucial role in tasks such as reading (Schotter et al., 2012). The area beyond the parafoveal region is referred to in this study as the peripheral region.

Based on previous research (Saunders & Woods, 2014), the latency of the gaze-contingent window was estimated to be around 20 ms. This estimate is based on the fact that the refresh rate of the monitor (60 Hz) is a limiting factor, and the fast response time of the BenQ monitor (Dispayspecifications.com, 2023).

Note that the edges of the circular dials were 318 and 342 pixels apart in the horizontal and vertical directions, respectively. Consequently, glancing between the dials in order to observe two dials simultaneously was not a viable strategy for performing the task. If one were to glance between two dials, one would only see a portion of those dials (a maximum of 91 pixels horizontally or 79 pixels vertically, while the diameter of each dial was 316 pixels).

### Experimental Design

The experiment was of a within-subject design, with two factors:

• Viewing condition. Each participant performed fourteen trials: seven with the videos in full-view condition and seven with the same seven videos in the gaze-contingent condition.
• Video number. The seven videos differed regarding the dial configuration (effort level).

### Experimental Procedure

Upon arrival, participants were informed about the aim of the experiment and read and signed the informed consent form. Next, participants faced the monitor and adjusted the seat height to comfortably position their heads on the support. The text displayed on the screen introduced the task to participants by stating they would be viewing 60 second videos featuring 6 dials with different pointer speeds. It further explained that the dashed lines represented the thresholds and instructed participants to press the spacebar whenever they noticed a pointer crossing one of these thresholds. The instruction screen displayed an image of a dial with a threshold, along with a screenshot of the full-view condition and the gaze-contingent condition, similar to Figure 2.

Next, the eye tracker was calibrated using the standard EyeLink 9-point calibration procedure. Then, participants proceeded to two short training trials: 20 seconds with the full view and 20 seconds with the gaze-contingent view (lowest effort level, with the two highest-bandwidth dials in the middle). Participants were able to repeat the training session if they requested it or if the experiment supervisor deemed it necessary, for example, if the participant was staring at one dial or not pressing the spacebar. In total, one participant repeated one training trial, and another participant repeated two training trials.

Each participant then completed two blocks of trials, each consisting of seven 60 second videos. Nineteen participants began with a block of videos in full view, followed by a block featuring the gaze-contingent window, while the other 14 participants experienced the opposite order. The order of the videos in the full-view condition and the gaze-contingent condition was the same for each participant, but different between participants.

### Data Processing

The gaze data for the two eyes were first averaged. A margin of 100 ms was added before and after periods of missing gaze data because of blinks or looking away from the monitor, and these were then filled in by linearly interpolating between the preceding and subsequent available gaze values. Moreover, a median filter with a 100 ms interval was applied to the *x* and *y* gaze coordinates. Gaze velocity data were calculated and filtered with a Savitzky–Golay filter with order 2 and a frame size of 20 ms, and the saccade velocity threshold was set at 2000 pixels/s. The minimum fixation was set at 40 ms. A 420 × 420 pixel area around each dial was used to determine whether a participant glanced at that dial.

### Dependent Variables

The following measures were calculated. Note that Measures 1 to 6 are identical to those applied in Eisma et al. (2018), while Measure 7 was added to give deeper insight into participants’ event detection performance.

**1.** ***Glance rate (1/s)***: Number of times the participant fixated on a dial divided by the trial duration. Consecutive re-fixations on the same dial were not counted in the computation of the glance rate. This glance rate is thought to closely represent Senders’ et al. (1966, 1983) primary dependent variable, which he referred to as “fixation frequency,” “sampling frequency,” or “frequency of observation.” In his studies, Senders (1983) assessed the directions of eye movements based on the inspection of 12 Hz film recordings, an approach that allowed him to infer at which dial the participant was looking.
**2.** ***Attention on dial (% of time)***: Percentage of video time for which the participants’ eyes were on a dial. This measure, also known as the net dwell time percentage, was calculated from the gaze point data, that is, before filtering for fixations and saccades.
**3.** ***Mean glance duration (s)***: The duration of attention on the dial in seconds (as used in Measure 2) divided by the number of glances on the dial (as used in Measure 1). The mean glance duration is thought to correspond to Senders’ (1983) definition of “duration of fixation.” Because consecutive re-fixations on the same dial were grouped (i.e., not counted as separate fixations), the mean glance duration is longer than the mean fixation duration as used in modern literature (which is typically around 250 ms).
**4.** ***Slope of glance rate***: Slope of a least-squares linear fit between the glance rate of the dial (Measure 1) and dial bandwidth (*n* = 6). Senders and the replication study by Eisma et al. (2018) found slopes of 0.64 and 0.61, respectively, that is, higher bandwidth dials received more attention than lower bandwidth dials. The slope is dimensionless due to the fact that it quantifies the relationship between glance rate (measured in 1/s or Hz) and dial bandwidth (also measured in Hz), resulting in a unit cancellation that yields a dimensionless ratio (Hz/Hz).
**5.** ***Slope of attention on dial (%/Hz)***: Slope of a least-squares linear fit between the attention on the dial (Measure 2) and dial bandwidth (*n* = 6). Senders and the replication study by Eisma et al. (2018) found slopes of 44.6 and 41.1%/Hz, respectively.
**6.** ***True-positive score (%)***: Participants were tasked to press the spacebar whenever any of the six pointers crossed the threshold angle. The true-positive score represents the percentage of threshold crossings for which the participant pressed the spacebar. There were between 52 and 78 threshold crossings per video. A loop was run over the threshold crossing events of the video in chronological order. For each threshold crossing, the temporally closest spacebar press was selected, and if the absolute time difference between the moment of pressing the spacebar and the moment of the threshold crossing was smaller than 0.5 s, then that threshold crossing was labeled a hit, and the spacebar press was excluded from being assigned to subsequent threshold crossings.
The justification for using a symmetrical time window, extending from negative (−0.5 s) to positive (+0.5 s) values rather than exclusively positive values, lies in the anticipatory nature of our spacebar-pressing task. In traditional reaction time research, the stimulus may be anticipated to some extent (Niemi & Näätänen, 1981), but fundamentally, it remains a response to a stimulus with a typical delay of about 200 ms. In our research, however, participants had the ability to continuously observe the pointer approach towards the threshold, which allowed them to engage in ongoing anticipation as opposed to merely reacting. In this sense, our experimental paradigm shares resemblances with the coincidence timing paradigm (Larson, 1989; Jensen, 2006). Although a minor delay is commonly observed, the distribution of response times around the threshold crossing moment is nearly symmetrical, with occurrences of participants pressing the spacebar both before and after the crossing (Eisma et al., 2018).

**7.** **False-positive score (#/s)**: The number of spacebar presses that were not assigned to a threshold crossing divided by the trial duration.

### Analysis

Scores on the dependent measures were compared between the gaze-contingent condition and the full-view condition. The analysis consisted of graphs depicting the relationship between dial bandwidth and sampling behavior, as in Senders (1964, 1983) and the replication study by Eisma et al. (2018). The current study also examined the effects of learning, that is, changes in the scores of the dependent measures as a function of trial number as well as within trials. Statistical tests used were paired-sample *t*-tests and repeated-measures ANOVAs.

## RESULTS

### Main Results

**Figure 3.**
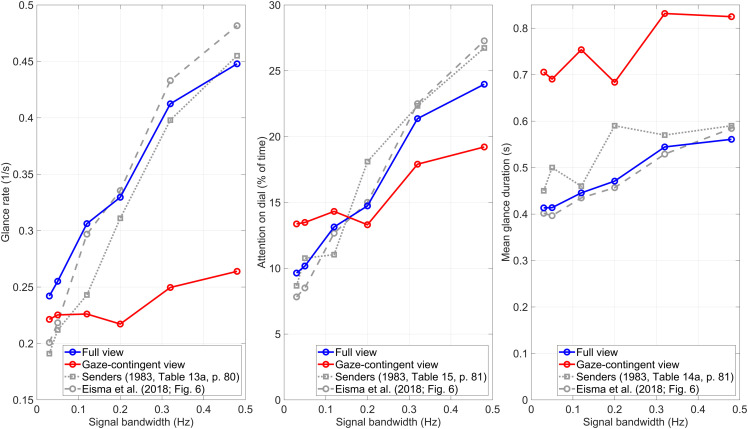
Glance rate, attention on dial, and mean glance duration as a function of the signal bandwidth of the dial. The gray lines correspond to findings from the literature.

Figure 3 shows results for three measures as a function of bandwidth: glance rate (left panel), attention on dial (middle panel), and mean glance duration (right panel). It can be seen that the results for the full-view condition (indicated in blue) closely match the original findings of Senders and the replication by Eisma et al. (2018) (gray lines).

For the gaze-contingent window (shown in red), participants distributed their attention less effectively between the six dials, as indicated by the substantially smaller (yet still positive) glance-rate slope (left panel) and attention-on-dial slope (middle panel) compared to the full-view condition (shown in blue). That is, while it would be desirable that high-bandwidth dials receive more attention than low-bandwidth dials, this ideal trend was considerably less apparent when peripheral vision was blocked. Figure 3 (right panel) also shows that with the gaze-contingent window, the mean glance duration was longer than the full-view condition.

**Figure 4.**
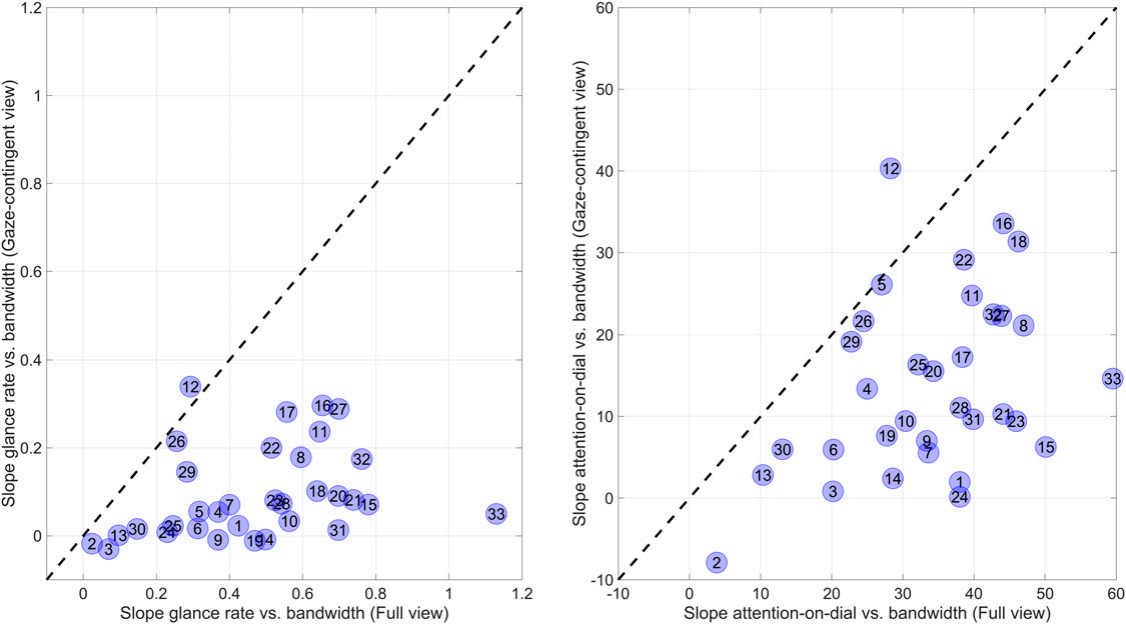
Slope of glance rate versus bandwidth (left panel) and percentage of attention-on-dial versus bandwidth (%/Hz), for the 33 participants individually. A higher slope is indicative of a more effective attention distribution across the six dials.

The attention distribution across the six dials as a function of bandwidth is further illustrated in Figure 4. Here, each marker represents the slope of the linear fit of an individual participant. For the glance rate (left panel), the mean slope was 0.47 (*SD* = 0.24) for the full view condition and 0.095 (*SD* = 0.105) for the gaze-contingent condition, *t*(32) = 9.28, *p* < .001. For the attention on dial (right panel), the mean slope was 33.6%/Hz (*SD* = 12.1) for the full-view condition and 13.9%/Hz (*SD* = 11.0) for the gaze-contingent condition, *t*(32) = 8.84, *p* < .001. Figure 4 makes clear that the effects of the gaze-contingent window on attention distribution were strong: only a few participants managed to distribute their attention in the gaze-contingent condition similarly to the full-view condition. To illustrate, the maximal glance-rate slope was 0.34 (Participant 12), which is still below the average of 0.47 for the full-view condition.

**Figure 5.**
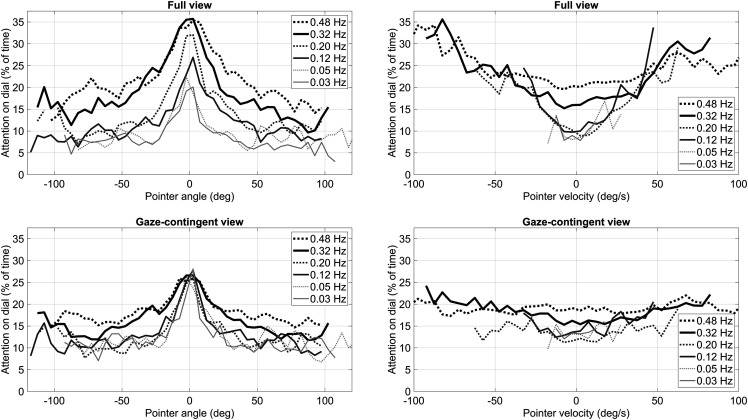
Percentage of time that attention is on the dial for a given pointer angle (in 5° increments) (left panels) and percentage of time that attention is on the dial for a given momentary pointer velocity (in 5°/s increments) (right panels). The results in this figure are based on all videos of all participants. Only data points for which at least 2 s of video data were available are shown.

Eisma et al. (2018) provided strong evidence for the so-called conditional sampling, that is, participants were likely to glance at a dial when the pointer was moving fast or when the pointer was close to the dial’s threshold angle. Figure 5 repeats the analysis of Eisma et al. by depicting the attention directed toward dials as a function of the momentary condition of the dial. More specifically, Figure 5 shows the percentage of the overall time that attention was on a dial for a momentary pointer angle relative to the threshold (left panels) and for a momentary pointer velocity (right panels). Figure 5 reveals considerable differences between the full-view condition and the gaze-contingent condition. Consistent with Eisma et al., for the full-view condition, participants were more likely to sample a dial when the dial was moving rapidly, as indicated by the U-shapes (right-top panel). This effect was not observed for the gaze-contingent view, as seen by the nearly flat lines (right-bottom panel). In regards to pointer angle, participants in the gaze-contingent condition were likely to glance at a dial when it was close to the threshold (left-bottom panel), but the effect was stronger in the full-view condition (left-top panel).

### Learning Effects

As mentioned in the Introduction, whether participants rely on expectancies (bandwidth) or peripheral cues depends on whether participants are able to form these expectancies. If expectancies were to be the primary driver of sampling behavior, then a learning effect can be expected. The importance of learning and experience was also emphasized by Senders: “*the experienced operator must have learned something about the statistics of the displayed signals given that H.O. [human operator] is able to look where looking is needed rather than where it is not. Any theory or model of the behaviour must include this characteristic*” (Senders, 1983, p. 17–18).

**Figure 6.**
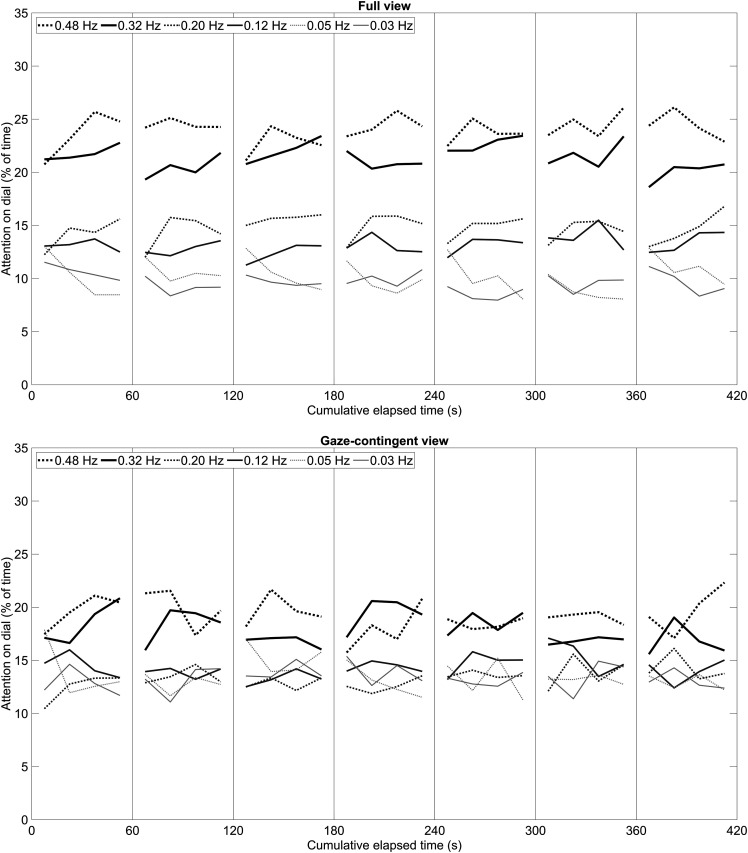
Percentage of time that participants had their eyes on a particular bandwidth dial as a function of the total elapsed video time. Each video lasted 60 s. The results are provided as averages per 15 s wide bin. Top figure: full-view condition; bottom figure: gaze-contingent condition.

Figure 6 shows the attention distribution of participants as a function of elapsed time in the full-view condition (top figure) and the elapsed time in the gaze-contingent condition (bottom figure). Visual inspection of the figures shows no clear learning trend; the attention-on-dial is similarly dispersed between dials at the beginning of the experiment compared to the end.

**Figure 7.**
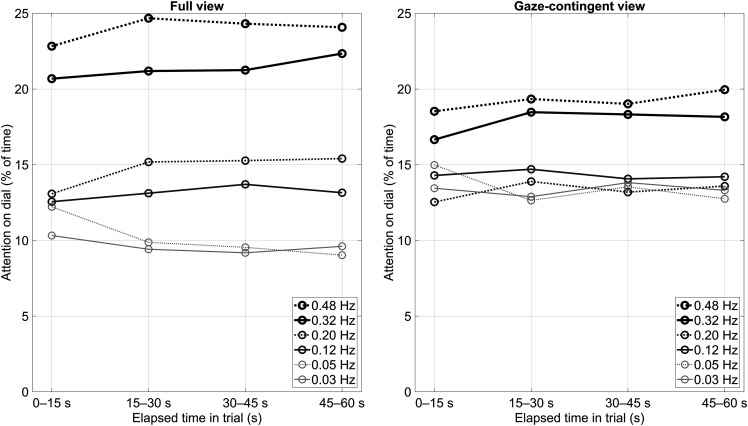
Attention on dial for the first, second, third, and fourth 15 s intervals of the 1 minute trials (averages of 7 videos). Left panel: full view; right panel: gaze-contingent view.

Each video featured a distinct arrangement of dials, with the high and low bandwidth dials situated in different positions. Hence, it could be argued that learning is expected to take place *within* individual trials, rather than from trial to trial. Accordingly, Figure 7 depicts the learning effects within the 1 minute trials. It can be seen that differences in attention-on-dial between the dials became more pronounced as the trial progressed. The degree of attention dispersion across the six dials was quantified by means of a linear fit between the attention-on-dial percentage and the dial’s bandwidth. The mean slopes of participants in the 0–15 and 45–60 s intervals of the full-view condition were found to be 28.4%/Hz and 35.7%/Hz, respectively, *t*(32) = 4.13, *p* < .001, while for the gaze-contingent condition, the mean regression slopes were 10.0%/Hz and 16.3%/Hz, respectively, *t*(32) = 3.38, *p* = .002.

**Figure 8.**
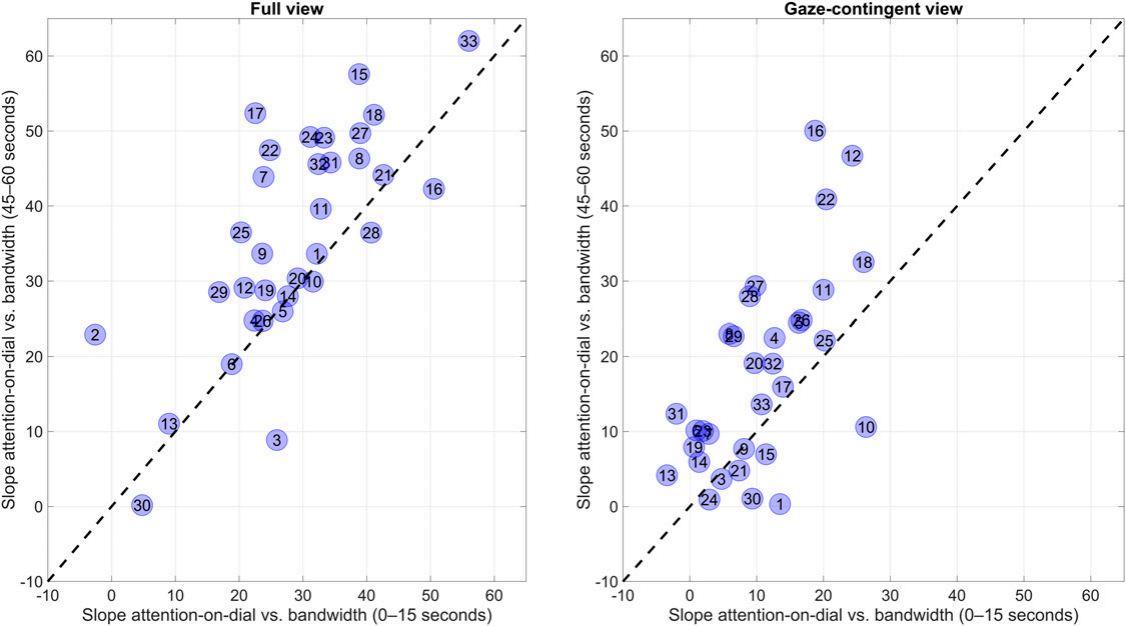
Slope of the percentage of attention-on-dial versus bandwidth (%/Hz), for the 33 participants individually, for the first and last 15 seconds of the 1 minute trials. A higher slope is indicative of a more effective attention distribution across the six dials.

Figure 8 depicts the slopes per participant for the 45–60 s interval compared to the 0–15 s interval. It can be seen that the majority of participants exhibited an increase in slope, as evidenced by markers lying above the diagonal line. In summary, there were statistically significant learning effects in attention distribution within the trials, both for full-view as well as the gaze-contingent view. Thus, at the start of the trials, participants tended to sample the dials in a relatively random manner, while by the end of the trial, they paid more attention to the higher bandwidth dials and less to the lower bandwidth dials.

In terms of the true-positive score, some learning was observed for the gaze-contingent condition. Specifically, in the first trial, participants detected 29% of threshold crossings, and in the last trial, this had increased to 34%, which is a statistically significant effect (as shown in Table 1). No significant learning effect was found for the full-view condition. A possible explanation for the nonsignificant effect may be the limited sample size and the blocked design of the experiment, where 19 participants began with the full-view condition and 14 with the gaze-contingent condition. This differs from the study by Eisma et al. (2018) where all participants only underwent the full-view condition and viewed longer videos (90 s) compared to the current study which used 60 s videos.

**TABLE 1:** Mean and *SD* of the True-Positive Score (%) as a Function of Presentation Order for the Seven Videos

	Full View	Gaze-Contingent View	Full View—Eisma et al. (2018)^a^
First	47.36 (13.11)	28.95 (8.89)	47.52 (8.76)
Second	46.40 (12.57)	29.31 (9.34)	48.46 (8.54)
Third	45.59 (13.23)	29.88 (9.14)	49.40 (9.14)
Fourth	47.87 (12.14)	30.84 (8.97)	48.31 (9.10)
Fifth	45.99 (12.94)	33.16 (9.07)	49.82 (8.11)
Sixth	48.34 (13.56)	31.74 (7.98)	51.34 (8.42)
Seventh (last)	46.26 (13.17)	33.62 (7.96)	51.17 (8.63)
Repeated-measures ANOVA	*F*(6,192) = 0.94, *p* = .464, partial η^2^ = 0.029	*F*(6,192) = 3.75, *p* = .001, partial η^2^ = 0.105	*F*(6,492) = 5.44, *p* < .001, partial η^2^ = 0.062

^a^These numbers differ slightly from Eisma et al. (2018) (<0.1%) because the code in Eisma et al. (2018) contained a minor mistake.

However, the overall true-positive score in the full-view condition was considerably higher (averaging at 46.8%, *SD* = 11.7%) compared to the gaze-contingent condition (averaging at 31.1%, *SD* = 7.2%), *t*(32) = 14.1, *p* < .001. On the other hand, the number of false positives per second, averaged across participants, was 0.117 (*SD* = 0.155) for the full-view condition, while it was only 0.059 (*SD* = 0.058) for the gaze-contingent condition, *t*(32) = 3.23, *p* = .003. These findings suggest that participants in the full-view condition applied a more liberal decision criterion. That is, in the full-view condition, participants were more active in pressing the spacebar (*M* = 0.592, *SD* = 0.255) compared to the gaze-contingent condition (*M* = 0.374, *SD* = 0.124), *t*(32) = 8.49, *p* < .001.

### Additional Analysis—Effort

Apart from replicating Senders’ (1964) work, Eisma et al. (2018) identified a role of effort, that is, one of the components of Wickens and McCarley’s (2008) SEEV model besides expectancy and salience. More specifically, Eisma et al. (2018) found that for low-effort videos (with the high-bandwidth dials placed in the center), participants distributed their attention more effectively as compared to high-effort videos (with high-bandwidth dials placed at the edges). We examined whether these results replicated in the current experiment.

Table 2 provides the regression slopes for each video separately, sorted on effort level. It can be seen that when the fast-moving dials were placed centrally (Level 1 effort), participants distributed their attention more in accordance with the Nyquist–Shannon sampling theorem, that is, with greater slope, than when the fast-moving dials were placed at the edges (Level 7 effort). This trend was statistically significant for the full-view condition, *F*(6, 192) = 33.45, *p* < .001, partial η^2^ = 0.511, as well as for the gaze-contingent condition, *F*(6, 192) = 13.90, *p* < .001, partial η^2^ = 0.303.

**TABLE 2:** Linear Fit for Bandwidth (W) as a Function of Mean Glance Rate (GR) for the Seven Different Videos. Also Shown are Results From Eisma et al. (2018)

	Full View	Gaze-Contingent View	Full View—Eisma et al. (2018)
Level 1 (lowest)	*GR* = 0.15 + 0.83 *W*	*GR* = 0.16 + 0.29 *W*	*GR* = 0.11 + 1.00 *W*
Level 2	*GR* = 0.20 + 0.69 *W*	*GR* = 0.21 + 0.15 *W*	*GR* = 0.16 + 0.84 *W*
Level 3	*GR* = 0.24 + 0.49 *W*	*GR* = 0.21 + 0.14 *W*	*GR* = 0.21 + 0.65 *W*
Level 4	*GR* = 0.27 + 0.35 *W*	*GR* = 0.23 + 0.02 *W*	*GR* = 0.23 + 0.50 *W*
Level 5	*GR* = 0.28 + 0.28 *W*	*GR* = 0.23 + 0.04 *W*	*GR* = 0.26 + 0.38 *W*
Level 6	*GR* = 0.24 + 0.44 *W*	*GR* = 0.21 + 0.06 *W*	*GR* = 0.18 + 0.66 *W*
Level 7 (highest)	*GR* = 0.29 + 0.21 *W*	*GR* = 0.24 - 0.04 *W*	*GR* = 0.23 + 0.44 *W*

## DISCUSSION

This study examined the effects of a gaze-contingent window on a human’s ability to effectively distribute attention across a bank of randomly moving dials, and on threshold crossing detection performance. While Senders (1964, 1983) argued in his works that humans likely distribute their attention based on signal bandwidth (expectancy), our results suggest that bandwidth is not the full explanation for the positive correlation between signal bandwidth and attention allocation time.

In the current experiment, participants failed to distribute their attention effectively across the dials (i.e., according to Senders’ normative models of attention distribution) when peripheral vision was blocked. The gaze-contingent window impaired the peripheral detection of pointers that moved at a high velocity (see Figure 5, right panels). A likely explanation for this phenomenon is that pointer velocity, being the derivative of pointer angle, can change abruptly and therefore represent a salient cue. Even though peripheral vision was unavailable, participants in the gaze-contingent condition were still able to direct their eyes to pointers that were close to the threshold (Figure 5, left panels). One possible explanation is that if participants observe that a pointer is close to the threshold, they may be more inclined to keep their eyes on that dial, while if they observe that a pointer is far away from the threshold, they may be inclined to fixate on another dial. Furthermore, pointer angle may be less of a salient cue than pointer velocity, since it requires a comparison with the threshold angle, something that likely requires the use of foveal vision. In summary, our findings suggest that the high correlation between signal bandwidth and attention allocation may, in part, be explained as an epiphenomenon of salient motion cues that are perceived using peripheral vision.

Further evidence of the importance of peripheral vision came from the fact that participants had longer glance times on the dials when peripheral vision was unavailable. A possible explanation is that, without access to peripheral vision, participants had less incentive to look away toward another dial. Thus, without peripheral vision, participants adopted a more passive monitoring style, as indicated by a prolonged attention span on individual dials resulting in a reduced number of scans between dials. Participants in the gaze-contingent condition also exhibited fewer false-positive spacebar presses than in the full-view condition, suggesting a more conservative decision threshold and overall more passive behavior. Previous studies using similar gaze-contingent paradigms concur that fixation durations are longer when only (para)foveal information is available as compared to having a full view of the scene (Bertera & Rayner, 2000; David et al., 2019; De Winter et al., 2023; Loschky & McConkie, 2002; Nuthmann, 2014).

The present findings do not imply that humans do not, or cannot, form expectancies about dial bandwidth. Some learning effects were observed, both within trials (Figures 7 and 8) and in terms of threshold-crossing detections across trials (Table 1). It seems plausible that these expectancies can be strengthened over time and that, given enough exposure to the task, sampling behavior would become less dependent on peripheral vision and more based on learned statistical properties of the pointer signals. In his work, Senders let participants perform the dial-monitoring task for up to 30 hours (Senders, 1983). However, the present findings show, like Eisma et al. (2018), that Senders’ findings can be replicated without much practice, and that salience is a strong contributor to the association between bandwidth and attention.

The present findings resonate with the debate on the role of top-down versus bottom-up attention (e.g., Theeuwes, 2010) and suggest that in the current dial monitoring task, bottom-up saliency cues have a crucial role. Our observations are consistent with research in which we found that participants were hardly able to remember the pointer angles immediately after the trial had ended (De Winter et al., 2019), suggesting that they performed the dial-monitoring task “automatically” without trying to reconstruct the signal. It is possible that the task was too abstract and short-lasting to form mental models, as may occur in more complex tasks such as car driving, in which the appearance of objects (hazards) is anticipatable from contextual cues (e.g., Yamani et al., 2022). To gain a deeper understanding of a potential role of expectancy in dial monitoring, it would be useful to repeat the study, but with multiple hours of practice spread over several days or weeks. In such a study, it would be important to use only one effort configuration, as this would allow the participant to form valid expectations about the behavior of the dials.

Our findings have important implications for the design of displays in practical applications. It is often assumed that operators must gain experience and form appropriate mental models, such as those of automated driving systems, in order to safely control those systems (Forster et al., 2019; Larsson et al., 2014). Although the importance of expectancy formation is not to be denied, the present study suggests a key role of salient motion cues that are understood almost instantly. This study further illustrates that attention distribution is more effective when visual scanning effort is low. These findings provide empirical support for display design recommendations previously formulated by Wickens and McCarley (2008): “*we prescribe that display designers should (a) correlate salience with value so that important events will be likely to capture attention; and (b) correlate distance, or effort, between two AOIs inversely with their joint expectancy so that sources of high bandwidth are close together, requiring minimal attention travel*” (p. 58). Thus, it may be argued that critical events should be sufficiently salient and that any interface should be designed so that even without any experience, people can understand it (Rahman, 2012). Applying these principles to an automated driving system, a head-up display could be used to highlight potential hazards and system limits, making it clear when and how the driver should regain control. By incorporating salient motion cues, such as spinning or moving elements, the display can effectively capture the attention of nonattentive drivers. This approach would improve the driving experience by making it more intuitive while reducing dependence on the driver’s experience and situation awareness.

## KEY POINTS

- A replication study of seminal dial-monitoring research by Senders was performed.
- A spotlight gaze-contingent window was used in half of the trials.
- Dial speed, as detectable using peripheral vision, attracts attention.
- Humans base their sampling on salience and bandwidth, rather than solely bandwidth.
